# 17,20S(OH)_2_pD Can Prevent the Development of Skin Fibrosis in the Bleomycin-Induced Scleroderma Mouse Model

**DOI:** 10.3390/ijms22168926

**Published:** 2021-08-19

**Authors:** Monica L. Brown Lobbins, Imara-Safi O. Scott, Andrzej T. Slominski, Karen A. Hasty, Sicheng Zhang, Duane D. Miller, Wei Li, Tae-Kang Kim, Zorica Janjetovic, Tejesh S. Patel, Linda K. Myers, Arnold E. Postlethwaite

**Affiliations:** 1 Departments of Pediatrics, University of Tennessee Health Science Center, Memphis, TN 38163, USA; lmyers@uthsc.edu; 2 Departments of Medicine, University of Tennessee Health Science Center, Memphis, TN 38163, USA; imara.hoyte@duke.edu (I.-S.O.S.); apostlelet@uthsc.edu (A.E.P.); 3 Departments of Dermatology, University of Alabama at Birmingham, 1720 2nd Avenue South, Birmingham, AL 35294, USA; aslominski@uabmc.edu (A.T.S.); tkkim4567@gmail.com (T.-K.K.); zjanjetovic@uabmc.edu (Z.J.); 4 O’Neal Comprehensive Cancer Center, University of Alabama at Birmingham, 1824 6th Ave., Birmingham, AL 35294, USA; 5 Birmingham VA Medical Center, 700 19th Street S., Birmingham, AL 35233, USA; 6 Departments of Orthopedic Surgery and Biomedical Engineering, University of Tennessee Health Science Center, Memphis, TN 38163, USA; khasty@uthsc.edu; 7 VA Medical Center, 1030 Jefferson Ave, Memphis, TN 38104, USA; 8 Department of Pharmaceutical Sciences, University of Tennessee Health Science Center, 881 Madison Ave, Memphis, TN 38163, USA; szhang71@uthsc.edu (S.Z.); dmiller@uthsc.edu (D.D.M.); wli@uthsc.edu (W.L.); 9 Department of Dermatology, University of Tennessee Health Science Center, Memphis, TN 38163, USA; tpatel3@uthsc.edu

**Keywords:** vitamin D, scleroderma, TGF-β1, bleomycin model of fibrosis, collagen, cytokines

## Abstract

Systemic sclerosis (SSc; scleroderma) is a chronic fibrotic disease involving TGF-β1. Low serum vitamin D (vit D) correlates with the degree of fibrosis and expression of TGF-β1. This study was designed to determine whether the noncalcemic vit D analog, 17,20S(OH)_2_pD, suppresses fibrosis and mediators of the TGF-β1 pathway in the bleomycin (BLM) model of fibrosis. Fibrosis was induced into the skin of female C57BL/6 mice by repeated injections of BLM (50 μg/100 μL) subcutaneously. Mice received daily oral gavage with either vehicle (propylene glycol) or 17,20S(OH)_2_pD using 5, 15, or 30 μg/kg for 21 days. The injected skin was biopsied; analyzed histologically; examined for total collagen by Sircol; and examined for mRNA expression of MMP-13, BMP-7, MCP-1, Gli1, and Gli2 by TR-PCR. Spleen was analyzed for lymphocytes using flow cytometry. Serum was analyzed for cytokines using a multiplexed ELISA. Results showed that all three doses of 17,20S(OH)_2_pD suppressed net total collagen production, dermal thickness, and total collagen content in the BLM fibrosis model. 17,20S(OH)_2_pD also increased MMP-13 expression, decreased MCP-1 and Gli-2 expression in vivo, and suppressed serum levels of IL-13, TNF-α, IL-6, IL-10, IL-17, and IL-12p70. In summary, 17,20S(OH)_2_pD modulates the mediators of fibrosis in vivo and suppresses total collagen production and dermal thickness. This antifibrotic property of 17,20S(OH)_2_pD offers new therapeutic approaches for fibrotic disorders.

## 1. Introduction

Systemic sclerosis (SSc; scleroderma) is a chronic disease characterized by extensive fibrosis of the skin and internal organs. Pathogenesis of this disease was reviewed by Pattanaik et al. [1] and includes autoimmunity, fibroblast activation, increased deposition of collagen type I (CI) and III (CIII), and decreased collagen degradation by matrix metalloproteinases (MMP). Collagen regulation is tightly controlled by the surrounding extracellular matrix (ECM) [2,3]. TGF-β1 contributes to fibrosis by promoting epithelial-to-mesenchymal transition (EMT), transitioning fibroblasts to a myofibroblast phenotype, contributing to conversion of adipocytes to fibroblasts, and by promoting fibroblast chemotaxis and the synthesis of collagen and fibronectin [3,4,5,6,7,8,9,10,11].

Animal models of fibrosis are used to study SSc disease pathogenesis and can mimic some pathophysiologic characteristics of the disease. Using the C57BL/6 murine model, cutaneous fibrosis can be induced by injecting bleomycin (BLM) subcutaneously into the same skin site on alternate days [12]. This model mimics early inflammatory changes in SSc with fibrosis limited to the area of injection [13].

In patients with SSc, low serum vit D correlates with the increased expression of TGF-β1 [14], degree of fibrosis, disease severity, and mortality [15,16,17,18]. Studies using 1,25 (OH)_2_D_3_ or a vit D analog to treat and reverse fibrosis have been previously reported [19,20,21]. In vivo, 1,25(OH)_2_D_3_ decreases the expression of TGF-β1, type I collagen, and plasminogen activator inhibitor-1 (PAI-1), which contributes to ECM homeostasis [22]. The vit D analog, paricalcitol, inhibited TGF-β signaling in cultured human normal fibroblasts, preventing upregulation of *col1a1* and production of collagen protein [23]. Topical treatment with calcipotriol decreased skin fibrosis and hydroxyproline content in the BLM model of fibrosis [19].

Current physiologic dosing of vit D supplementation has been successful only in correcting the deficiency of vit D without reversing fibrosis. Doses needed to reach pharmacologic levels to treat autoimmune disorders such as SSc and to have an impact on disease morbidity are likely not attainable with 1,25 (OH)_2_D_3_ due to its hypercalcemic risk [24,25,26]. We have previously published data showing that noncalcemic analogs of vit D with a full side chain including 20-hydroxyvitamin D_3_ [20(OH)D_3_] [27,28,29,30] and short-chain analogs, such as 17,20R(OH)_2_pD and 17,20S(OH)_2_pD, have antifibrotic properties and can inhibit TGF-β1-induced net collagen production, without inducing calcemia [22,23,24,25,26,27,28,29,30,31]. Molecular modeling using the crystal structure demonstrated that 20(OH)D_3_, 17,20R(OH)D_2_, and 17,20S(OH)D_2_ bind to the vit D receptor (VDR) [31,32,33,34]. We previously showed that 17,20R(OH)_2_pD and 17,20S(OH)_2_pD can suppress TGF-β1-driven collagen and hyaluronan production in vitro by human normal dermal fibroblasts [35].

In the present study, we demonstrate 17,20S(OH)_2_pD administered by oral gavage decreases the development of dermal fibrosis and loss of subcutaneous adipose tissue, and affects the expression of key players in the TGF-β1 pathway in the BLM-induced scleroderma model of fibrosis

## 2. Results

### 2.1. 17,20S(OH)_2_pD Decreases Dermal Thickness

**Figure 1:**
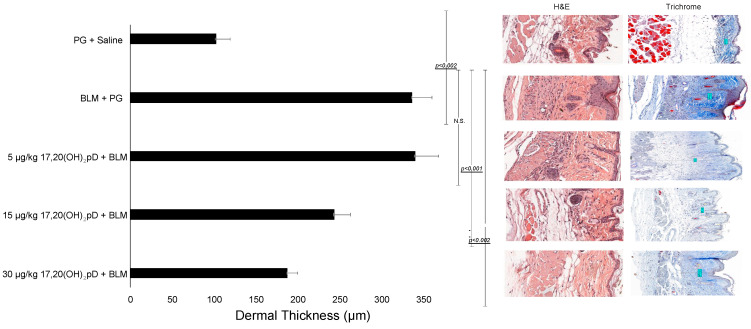
17,20S(OH)_2_pD decreases dermal thickness in BLM model of fibrosis. C57BL/6 mice (*n* = 6 per group) received oral gavage daily with either vehicle (100 µL propylene glycol 1:5 diluted PG) or 17,20(OH)_2_pD at either 5, 15, or 30 µg/kg per 100 µL of 1:5 diluted PG for 21 days. The skin was injected s.c. with either bleomycin 50 µg per 100 µL saline or 100 µg saline daily for 21 days, then extracted for histology and collagen synthesis. *p* values were determined by ANOVA; *p* < 0.05, significant. Left panel: quantification of the dermal thickness; middle panel: H&E stained sections; right panel: Trichrome stained sections. All are 20× magnification.

Figure 1 shows attenuation of dermal thickness by 17,20S(OH)_2_pD induced by BLM injections with quantitative data and representative histology, both H&E and trichrome stains. Sectioned skin biopsies of the BLM injection site from mice treated with 17,20S(OH)_2_pD showed a significant decrease in dermal thickness compared to skin biopsies from vehicle control mice (Figure 1). Sections of skin from the BLM injection site from mice treated with vehicle showed increased collagen deposition and decreased adipose tissue in the subcutaneous layer compared to saline-injected mice (Figure 1). Treatment with 17,20S(OH)_2_pD not only prevented the development of increased dermal thickness at the BLM injection site but also allowed the maintenance of subcutaneous adiposity (Figure 1).

### 2.2. 17,20S(OH)_2_pD Suppresses Total Collagen Content in the BLM Fibrosis Model

**Figure 2:**
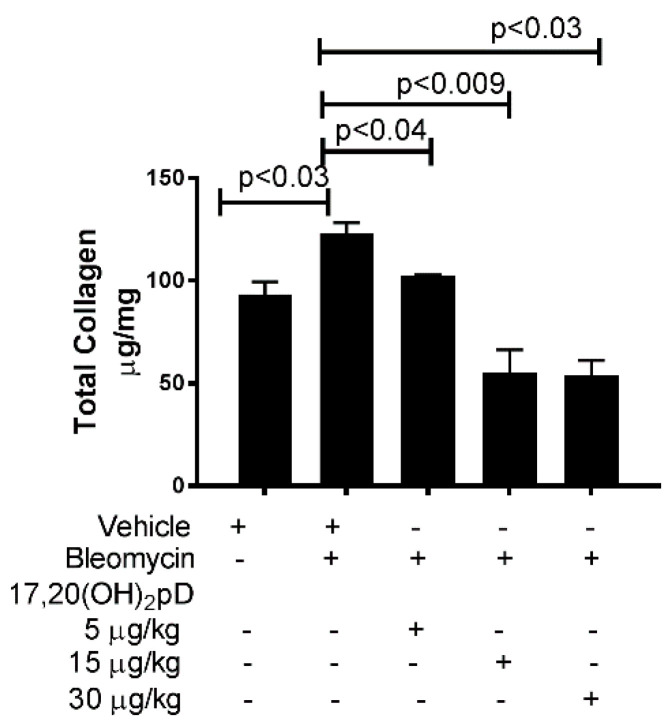
17,20S(OH)_2_pD suppresses total collagen production in the BLM model of fibrosis. C57BL/6 mice (n = 6 per group) treated via gavage with either 17,20(OH)_2_pD at doses of 5, 15, and 30 μg/kg or vehicle (propylene glycol) while simultaneously receiving s.c. injections of bleomycin for 21 days. Injected skin was analyzed for total collagen using Sircol collagen assay. Results are shown as mean ± SEM and *p* values were determined by ANOVA by comparing vehicle (PG) + BLM with those from PG + saline or 17,20(OH)_2_pD + BLM. + added to culture; - not added to culture.

We assessed the effect of 17,20S(OH)_2_pD on collagen content at the site of skin that received repeated injections of BLM. Mice that received oral gavage with either 17,20S(OH)_2_pD 5, 15, or 30 μg/kg in contrast to mice that received oral gavage with vehicle control (PG) had reduced levels of total collagen in the skin at the site of BLM injection. We observed a significant reduction in net total collagen levels at the BLM injection site with increasing doses of 17,20S(OH)_2_pD (Figure 2). 17,20S(OH)_2_pD, at the highest dose administered (30 μg/kg), had no effect on calcium levels in serum (Ca^++^ 7 ± 1.30 mg/dL) compared to vehicle-treated mice (7 ± 0.5 mg/dL; *p* = 0.85).

### 2.3. 17,20S(OH)_2_pD Modulates the Mediators of the TGF-β1 Pathway

**Figure 3:**
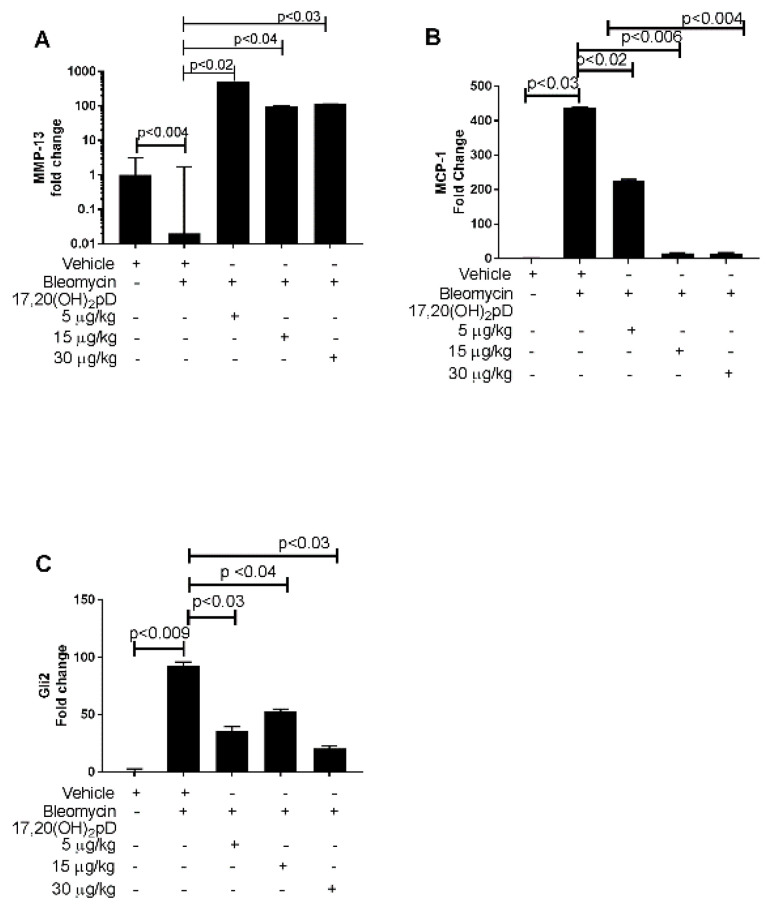
17,20S(OH)_2_pD modulates the mediators of the TGF-β1 pathway. C57BL/6 mice (n = 6 per group) received oral gavage daily with either vehicle (100 µL propylene glycol 1:5 diluted PG) or 17,20(OH)_2_pD at either 5, 15, or 30 µg/kg per 100 µL of 1:5 diluted PG for 21 days. The skin was injected s.c. with either bleomycin 50 µg per 100 µL saline or 100 µg saline daily for 21 days, then injected skin was extracted for total RNA and analyzed for MMP-13 (**A**), MCP-1 (**B**), and Gli 2 (**C**). Results are shown as mean ± SEM and *p* values were determined by ANOVA by comparing vehicle (PG) +BLM with those from PG + saline or 17,20(OH)_2_pD + BLM. + added to culture; - not added to culture.

A decrease in the synthesis of MMPs is one of several mechanisms that contribute to fibrosis in SSc. MMP-13 is a well-known collagenase, acknowledged for its involvement in the pathogenesis of fibrotic disorders [36]. We examined whether treatment of mice with 17,20S(OH)_2_pD would increase MMP-13 mRNA expression at the site of BLM injection in the skin. Compared to levels of MMP-13 mRNA in the site of injection of saline in control mice, MMP-13 mRNA levels in the site of BLM injection in the BLM-treated mice significantly decreased (Figure 3A). Oral gavage treatment of the BLM-treated mice with either of the three doses of 17,20S(OH)_2_pD compared to oral gavage with vehicle was associated with an upregulation of MMP-13 mRNA expression in the skin at the BLM injection site (Figure 3A).

We next examined whether 17,20S(OH)_2_pD affected the level of mRNA of MCP-1, a chemokine that can promote fibrosis. BLM increased MCP-1 mRNA expression in BLM-injected mice compared to vehicle-treated mice who received s.c. injections of saline. 17,20S(OH)_2_pD decreased the expression of MCP-1 mRNA at all three doses (Figure 3B). 

We also assessed effect of 17,20S(OH)_2_pD on the mRNA levels of fibrogenic mediators BMP-7, Gli-1, and Gli-2 mRNA. Bleomycin increased the expression of BMP7, Gli-1, and Gli-2 mRNA in vehicle-treated mice. The increased mRNA level of Gli-2 induced by BLM at the skin injection site was reduced by 17,20S(OH)_2_pD treatment (Figure 3C). 17,20S(OH)_2_pD did not significantly lower the bleomycin stimulation of BMP-7 or Gli1 mRNA.

We examined the 17,20S(OH)_2_pD effect on splenic lymphocytes by flow cytometry. Excised spleens from treated mice were analyzed for percentages of CD19^+^ B-cells, CD3^+^ T-cells, CD4^+^, CD8^+^, and Foxp3 CD25^+^ lymphocytes. When gated on lymphocytes, BLM alone increased the percentages of CD19^+^ B-cells (58 ± 1.1 vs. 49 ± 1.2; *p* < 0.01), while the addition of 17,20S(OH)_2_pD at 30 μg/kg increased CD19^+^ B-cells to an even greater level (65 ± 1.4 vs. 58 ± 1.1; *p* < 0.0002). BLM alone decreased the percentages of CD3^+^ T-cells (18 ± 1.4 vs. 25 ± 1.0; *p* < 0.04) compared to vehicle-treated mice. However, the addition of 17,20S(OH)_2_pD increased the levels of CD3^+^ T-cells compared to BLM-treated mice, returning them closer to normal levels; (22 ± 1.4 vs. 18 ± 1.1; *p* < 0.02) when treated with 15 μg/kg. Culture with 17,20S(OH)_2_pD or BLM did not significantly affect Foxp3 CD25^+^ lymphocytes or CD4^+^/CD8^+^ ratios. 

### 2.4. 17,20S(OH)_2_pD Modulates Cytokines in the BLM Model of Fibrosis

We examined whether 17,20S(OH)_2_pD treatment of BLM-injected mice affected the serum expression of cytokines. Mice treated with s.c. BLM in contrast to mice treated with s.c. saline exhibited significant increase in serum levels of Eotaxin, G-CSF, IL-1β, IL-2, IL-3, IL-5, IL-12p70, KC, MCP-1, and MIP-1β, but reduced the basal serum levels of GM-CSF, IL-6, IL-10, IL-12p40, IL-13, or TNF-α. Administration of 15 μg/kg of 17,20S(OH)_2_pD to mice treated with s.c. BLM for 21 days by gavage significantly reduced serum levels of IL-3, IL-5, IL-6, IL-10, IL-12p70, IL-13, IL-17, KC, MCP-1, MIP-1β, and TNF-α, but had no significant change in serum levels of IL-1β and IL-2. Administration of 15 μg/kg of 17,20S(OH)_2_pD to mice treated with s.c. BLM significantly increased serum levels of IL-12p40 and reduced Eotaxin and GM-CSF to levels below the detection limit of the multiplex ELISA (Table 1). Serum levels of IFN-γ, IL-1α, IL-4, IL-9, MIP-1α, or RANTES were not affected by either BLM or 15 μg/kg of 17,20S(OH)_2_pD.

## 3. Discussion

Collagen overproduction is a hallmark feature of SSc. 17,20S(OH)_2_pD decreased the total collagen content of the skin at the site of BLM injection in mice. The suppression of collagen content exhibited a dose-dependent pattern. These findings are consistent with our previous study showing that 17,20S(OH)_2_pD decreased collagen synthesis in cultured human dermal fibroblasts stimulated with TGF-β1 [35].

Using the BLM model of fibrosis, vit D analog 22-oxacalcitriol (OCT) decreased dermal collagen bundle without influencing dermal thickness at the site of BLM injection [31]. In the current study, the skin at the site of injected BLM showed increased dermal fibrosis and reduction in subcutaneous adipose tissue compared with the skin at the site of repeated saline injection. The fibrosis also extended into the subcutaneous fat layer. These findings closely mirror the clinical presentation of SSc. Mice that were subjected to BLM injection and treated with 17,20S(OH)_2_pD had a significant reduction in skin fibrosis and maintenance of the subcutaneous fat layer. This study introduces evidence of BLM-induced dermal thickness reduction by 17,20S(OH)_2_pD. Reduction in dermal thickening is a crucial component of skin softening and overall mobility in patients with scleroderma.

The present study highlights the effect of 17,20S(OH)_2_pD on the mediators in the TGF-β1 pathway. MMP-13 expression is suggested to be a strong indicator of disease severity in fibrosing disorders. Lower expression of MMP-13 was found in patients with generalized morphea compared to normal controls [36]. There is an inverse correlation between serum MMP-13 levels and the degree of body area that is affected by fibrosis. In the present study, we found that 17,20S(OH)_2_pD at 5, 15 and 30 μg/kg increased MMP-13 expression in the BLM model.

MCP-1 is a chemokine that induces inflammation and fibrosis. Mice injected with BLM had a significant increase in MCP-1 production [32]. Yamamoto et al. identified the direct and indirect pro-inflammatory effects exerted by MCP-1 on fibroblast proliferation [33]. In the present study, our findings are consistent in that MCP-1 mRNA expression increased at the site of BLM skin injection and its expression was decreased in BLM injected mice treated with 17,20S(OH)_2_pD. The serum levels of MCP-1 increased in mice that received s.c. injections of BLM; 17,20S(OH)_2_pD decreased serum levels of MCP-1, although non-significantly.

BMP-7, an anti-fibrotic mediator, acts to antagonize the profibrotic effects TGF-β1 [37]. Current evidence regarding the attenuating effects of BMP-7 on fibrosis is unclear. Using murine dermal papilla cells, Bin et al. were able to show inhibition of the TGF-β1 pathway by BMP-7 [37]. Other studies suggest that BMP-7 is less effective at inhibiting TGF-β1 for both in vivo and in vitro BLM-induced skin and lung fibrosis models, suggesting that perhaps renal models show the best reduction in fibrosis by BMP-7 [38,39]. In the present study, BLM increased BMP-7 expression, and 17,20S(OH)_2_pD had no significant effect on BMP-7 expression in treated mice, suggesting that the 17,20S(OH)_2_pD effect on fibrosis in the BLM skin fibrosis murine model is not mediated by increased BMP-7 production.

Gli-1 and Gli-2 are transcription factors in the hedgehog pathway and are positive mediators of TGF-β1 signaling. Inhibiting Gli-1 transcriptional activity is correlated with decreased lung fibrosis and collagen production [40]. In the present study, BLM increased Gli-1 and Gli-2 but 17,20S(OH)_2_pD did not suppress Gli-1 production but suppressed Gli2 production at 5 and 30 μg/kg, suggesting that 17,20S(OH)_2_pD’s mechanism of TGF-β1 inhibition of fibrosis may involve portions of the hedgehog pathway.

Finally, we evaluated the effects of 17,20S(OH)_2_pD on various cytokines, some of which are known to be associated with scleroderma and fibrogenesis. IL-6, IL-13, IL-17, and TNF-α are cytokines recognized to be involved in the pathophysiology of scleroderma [40]. Several studies have implicated each of these cytokines in collagen production, and lung and systemic fibrosis [41,42,43,44,45,46,47,48]. In the present study, serum levels of IL-12p40, IL-12p70, IL-6, IL-13, IL-17, MCP-1, and TNF-α were decreased in mice treated with 17,20S(OH)_2_pD. 17,20S(OH)_2_pD modulated the serum expression of IL-6, IL-10, IL-13, IL-17, and TNF-α independent of the BLM effect on these cytokines, suggesting that 17,20S(OH)_2_pD, as a vit D, analog has properties that can alter these cytokines and effect fibrosis. Serum vascular endothelial growth factor (VEGF) is another critical mediator of inflammation known to play a key role in endothelial dysfunction and is increased in systemic lupus erythematosus patients [49]. Since vitamin D was shown to decrease VEGF in clinical trials, it will be important to determine how well 17,20S(OH)_2_pD decreases VEGF in SSc patients in future studies [50]. Moreover, since the immune system and microbiome are interconnected and vitamin D was shown to be critical for maintaining a healthy microbiome, it will be of interest to study how 17,20S(OH)_2_pD affects the microbiome in scleroderma and other autoimmune patients [51].

In conclusion, this study shows that 17,20S(OH)_2_pD modulates mediators of fibrosis in vivo, and suppresses total collagen production and dermal thickness, wherein a balance will favor a reduction in fibrosis and may offer a new therapeutic approach for treating scleroderma and other disorders of fibrosis.

Our study is limited in that it provides evidence of 17,20S(OH)_2_pD’s ability to prevent fibrosis and does not address 17,20S(OH)_2_pD’s ability to treat or regress established fibrosis. We will address this important issue in future studies using the experimental approach outlined by Akhmetishina et al. (Alfiya Akhmetshina et al. Oliver Distler, Jörg H. W. Distler; Pages: 219–224). This study is also limited in that it addresses 17,20S(OH)_2_pD’s ability to prevent skin fibrosis in the bleomycin scleroderma model but does not include a validation study that addresses its ability to prevent fibrosis in another scleroderma model. A second independent validation study using a separate scleroderma mouse model will be a project for future studies to address this limitation.

## 4. Materials and Methods

Experiments using mice were approved by the Institutional Animal Care and Use Committee at the University of Tennessee Health Science Center (project #18-092), approved 24 October 2018). Secosteroid 17,20S(OH)_2_pD was synthesized as previously described [29,30]. For oral gavage experiments in mice, 17,20S(OH)_2_pD was dissolved in propylene glycol (PG) (Cat. #P4347; Sigma Aldrich, St. Louis, MO, USA), which was then diluted with 4 parts sterile saline and brought to the desired concentration by adding 1:5 diluted PG (1 part PG + 4 parts sterile saline). Stock Bleomycin (500 μg/1000 μL) (Teva Parenteral Medicines Inc., Irvine, CA, USA) was diluted with IV sterile saline to a final concentration of 50 μg/100 μL. 

### 4.1. Induction of Skin Fibrosis in Mice

Female C57BL/6 mice, 6 weeks old, were purchased from Jackson Labs (Bar Harbor, ME, USA) and were maintained on a regular chow diet. Groups of mice (6 each) received oral gavage daily for 21 days with either vehicle (100 µL propylene 1:5 diluted PG) or 17,20S(OH)_2_pD at either 5, 15, or 30 µg/kg per 100 µL of 1:5 diluted PG. The skin was injected s.c. on the dorsolateral back with either bleomycin 50 µg per 100 µL saline or 100 µL saline daily within the same 1.5 cm^2^ area for 21 days. The oral gavage and bleomycin injections were performed on the same days. On Day 22, mice were euthanized and sections of biopsied skin were processed for histology, total collagen analysis, or real-time polymerase chain reaction (RT-PCR). Sections of spleen were analyzed for CD19^+^ B-cells, CD3^+^T cells, CD4^+^, CD8^+^, and Foxp3CD25^+^ lymphocytes by flow cytometry. Sera were analyzed for cytokines using the BIO-Plex Pro Mouse Cytokine 23-Plex panel (IL-1α, IL-1β, IL-2, IL-3, IL-4, IL-5, IL-6, IL-9, IL-10, IL-12p40, IL-12p70, IL-13, IL-17, eotaxin, G-CSF, GM-CSF, IFN-γ, KC, MCP-1, MIP-1α, MIP-1β, RANTES, and TNF-α; Cat. #M60-009RDPD; Bio-Rad, Hercules, CA, USA) and total calcium (Cat. #AB102505; Abcam, Cambridge, MA, USA).

### 4.2. Histology Analysis

Biopsied skin sections of the site where BLM or saline was injected were formalin-fixed and processed for histopathologic analysis using hematoxylin and eosin and trichrome staining. The quantitative analysis of the dermal thickness between different groups was assessed by light microscopy by two evaluators (ATS and AEP) using an Olympus microscope with 20× magnification. Three skin layers were included, and six sections were measured for each evaluation.

### 4.3. Quantitative Real-Time PCR

Total RNA was extracted from homogenized skin of mice using Trizol reagent (Invitrogen-Thermo Fisher Scientific, Waltham, MA, USA) and reversed-transcribed into cDNA using reverse transcriptase (Life Technologies-Applied Biosystems (ABI), Grand Island, NY, USA) for RT-PCR. RNAs were quantified using NanoDrop-2000 (Thermos, Franklin, NJ, USA). Fifteen nanograms of total RNA was used for these experiments. TaqMan gene assay for murine BMP-7 (Cat. No. Mm00432102_mL), COL1a1 (Cat. No. Mm00801666_gl), Gli1 (Cat. No. Mm 00494654_mL), Gli2 (Cat. No. Mm01293111_mL), MCP-1 (Cat. No. Mm00441242_mL), MMP-13 (Cat. No. Mm00439491_mL), beta actin (Cat. No. Mm00607939_sl; Applied Biosystems Life technologies, Grand Island, NY, USA). Beta actin was used as a housekeeping gene. Roche LC-480 was used to run PCR, and analysis was obtained with RQ manager (Life Technologies-ABI, Waltham, MA, USA). 17,20S (OH)_2_pD was normalized to β-actin (Applied Biosystems Life Technologies, Grand Island, NY, USA) mRNA expression levels. Data for 17,20S (OH)_2_pD treated mice are shown as fold change normalized to an endogenous reference (PG) vehicle control.

### 4.4. Analysis of Total Collagen

Sections of biopsied injected skin from each mouse were digested with pepsin ((0.1 mg/mL) and 0.5 M acetic acid) overnight at 4 °C to remove terminal nonhelical telopeptides and to release collagen into solution as previously described [52]. Total solubilized collagen was measured using a Sircol collagen assay kit (Bicolor Ltd., Carrickgergus, Co Antrim, UK) per the manufacturers protocol. Data are presented as the mean µg/mL ± SEM of collagen per each group.

### 4.5. Quantitation of Serum Calcium

A calcium assay kit was employed to quantitate the levels of Ca^++^ in mouse sera (Cat# AB102505; Abcam, Cambridge, MA, USA).

### 4.6. Flow Cytometry

Excised spleen tissue from C57BL/6-treated mice were washed with phosphate-buffered saline (PBS) and stained for CD3 (Alexa Fluor-700), CD4 (PE), CD8 (FITC), CD19 (APC), CD25 (PerCP-Cy7), and Foxp3 (Alexa Fluor-488) according to the manufacturer’s recommendations. All antibodies were obtained from BD Bioscience. A minimum of 10,000 cells were analyzed from each sample using BD LSR II flow cytometry (BD Bioscience) and analysis was performed using FlowJo v10.1 (Tree Star; Ashland, OR, USA).

### 4.7. Statistical Analyses

Data are presented as mean ± SE and analyzed with Student’s *t*-test for groups of two or ANOVA using Prism 4.00 (GraphPad, San Diego, CA, USA). Differences in values obtained with the vit D hydroxyl derivatives were compared with appropriate controls using ANOVA. *p* ≤ 0.05 was considered statistically significant.

## 5. Conclusions

In the current study, we showed that 17,20S(OH)_2_pD can: (1) suppress net total collagen synthesis, (2) modulate key mediators in the TGF-β1 pathway, and (3) alter expression of inflammatory cytokines in the bleomycin (BLM) model of skin fibrosis.

## Figures and Tables

**Table 1:** 17,20S(OH)_2_pD modulates cytokines in the BLM model of skin fibrosis.

**Condition**	**IL** **-1β**	**IL-2**	**IL-3**	**IL-5**	**IL-6**	**IL-10**
PG + Saline	20 ± 5	14 ± 4	9 ± 2	8.0 ± 3	11 ± 4	33 ± 18
PG + BLM	161 ± 25 *p* < 0.00002	32 ± 12 *p* < 0.006	14 ± 2 *p* < 0.004	11 ± 1.0 *p* < 0.04	12 ± 2 *p* = 0.3	47 ± 7 *p* = 0.13
BLM + 1720S (OH)_2_pD 15 μg/kg	157 ± 50 *p* = 0.5	30 ± 16 *p* = 0.4	10 ± 2 *p* < 0.02	9.4 ± 0.8 *p* < 0.02	7 ± 3 *p* < 0.007	20 ± 7 *p* < 0.002
**Condition**	**IL-12p40**	**IL-12p70**	**IL-13**	**IL-17**	**Eotaxin**	**G-CSF**
PG + Saline	36 ± 21	304 ± 80	337 ± 162	12 ± 3	571± 313	14 ± 4
PG + BLM	42 ± 15 *p* = 0.32	514 ± 135 *p* < 0.02	442 ± 27 *p* = 0.12	15 ± 3 *p* = 0.07	1510 ± 333 *p* < 0.0009	32 ± 12 *p* < 0.006
BLM + 1720S (OH)_2_pD 15 μg/kg	97 ± 49 *p* < 0.03	341 ± 67 *p* < 0.03	178 ± 52 *p* < 0.0002	12 ± 2 *p* < 0.04	undetectable	30 ± 16 *p* = 0.4
**Condition**	**KC**	**MCp -1**	**MIP-1α**	**TNF-α**	**GM-CSF**	
PG + Saline	9 ± 2	8.0 ± 3	30.1 ± 13	187 ± 66	39 ± 17	
PG + BLM	14 ± 2 *p* < 0.004	11 ± 1.0 *p* < 0.04	62 ± 24 *p* < 0.01	264 ± 75 *p* = 0.06	47 ± 17 *p* = 0.23	
BLM + 1720S (OH)_2_pD 15 µg/kg	10 ± 2 *p* < 0.02	9.4 ± 0.8 *p* < 0.02	7 ± 3 *p* < 0.007	137 ± 73 *p* < 0.01	undetectable	

## Data Availability

The datasets used and/or analyzed during the current study are available from the corresponding author on reasonable request.
